# 3-(4-Bromo­anilino)-3-(4-chloro­phen­yl)-1-phenyl­propan-1-one

**DOI:** 10.1107/S1600536811036932

**Published:** 2011-09-17

**Authors:** Mehrdad Pourayoubi, Zohreh Shobeiri, Giuseppe Bruno, Hadi Amiri Rudbari

**Affiliations:** a Department of Chemistry, Ferdowsi University of Mashhad, Mashhad 91779, Iran; b Dipartimento di Chimica Inorganica, Vill. S. Agata, Salita Sperone 31, Università di Messina, 98166 Messina, Italy

## Abstract

The asymmetric C atom in the title compound, C_21_H_17_BrClNO, is in a slightly distorted tetra­hedral environment and the NH unit adopts a *gauche* orientation with respect to the CO group. In the crystal, pairs of inter­molecular N—H⋯O hydrogen bonds form centrosymmetric dimers.

## Related literature

For background to β-amino ketones, see: Scettri *et al.* (2008 ▶). For related structures, see: Shobeiri *et al.* (2011 ▶); Zhang *et al.* (2008 ▶). For hydrogen-bond motifs and their graph-set notation, see: Bernstein *et al.* (1995 ▶).
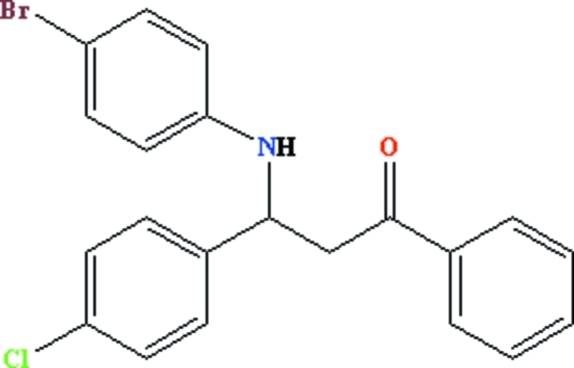


## Experimental

### 

#### Crystal data

C_21_H_17_BrClNO
*M* *_r_* = 414.72
Monoclinic, 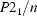

*a* = 10.6571 (4) Å
*b* = 17.2432 (6) Å
*c* = 10.8602 (4) Å
β = 113.571 (2)°
*V* = 1829.19 (12) Å^3^
*Z* = 4
Mo *K*α radiation
μ = 2.40 mm^−1^
*T* = 296 K
0.35 × 0.31 × 0.11 mm

#### Data collection

Bruker APEXII CCD diffractometer
Absorption correction: multi-scan (*SADABS*; Sheldrick, 2004 ▶) *T*_min_ = 0.589, *T*_max_ = 0.746
69312 measured reflections
3983 independent reflections
3274 reflections with *I* > 2σ(*I*)
*R*_int_ = 0.036

#### Refinement

*R*[*F* ^2^ > 2σ(*F* ^2^)] = 0.033
*wR*(*F* ^2^) = 0.084
*S* = 1.03
3983 reflections
230 parameters
H atoms treated by a mixture of independent and constrained refinement
Δρ_max_ = 0.71 e Å^−3^
Δρ_min_ = −0.86 e Å^−3^

### 

Data collection: *APEX2* (Bruker, 2005 ▶); cell refinement: *SAINT* (Bruker, 2005 ▶); data reduction: *SAINT*; program(s) used to solve structure: *SHELXS97* (Sheldrick, 2008 ▶); program(s) used to refine structure: *SHELXL97* (Sheldrick, 2008 ▶); molecular graphics: *SHELXTL* (Sheldrick, 2008 ▶) and *Mercury* (Macrae *et al.*, 2008 ▶); software used to prepare material for publication: *SHELXTL*.

## Supplementary Material

Crystal structure: contains datablock(s) I, global. DOI: 10.1107/S1600536811036932/qm2027sup1.cif

Structure factors: contains datablock(s) I. DOI: 10.1107/S1600536811036932/qm2027Isup2.hkl

Supplementary material file. DOI: 10.1107/S1600536811036932/qm2027Isup3.cml

Additional supplementary materials:  crystallographic information; 3D view; checkCIF report

## Figures and Tables

**Table 1:** Hydrogen-bond geometry (Å, °)

*D*—H⋯*A*	*D*—H	H⋯*A*	*D*⋯*A*	*D*—H⋯*A*
N1—H⋯O1^i^	0.81 (3)	2.23 (3)	2.992 (3)	156 (2)

Symmetry code: (i) 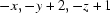.

**Fig. 1.**
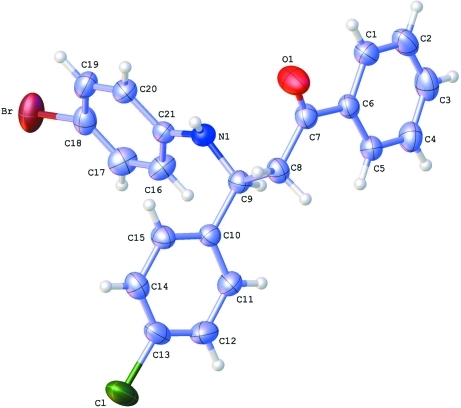
An ORTEP-style plot of title compound with labeling. Ellipsoids are given at the 50% probability level.

**Fig. 2.**
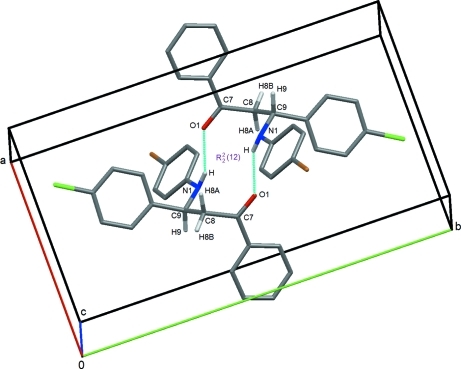
Part of the crystal packing of the title compound showing a centrosymmetric H-bonded (dashed lines) dimer. Only H atoms involving in hydrogen bonds are shown.

## supplementary crystallographic information

### Comment

β-Amino ketones, of the formula [*R*^1^]CH[NH*R*^2^][CH_2_C(O)*R*^3^], such as the title compound have attracted attention because of their roles as important intermediates for the synthesis of natural products and chiral auxiliaries (Scettri *et al.*, 2008). In the previous work, the structure determination of 3-(4-bromophenylamino)-1-phenyl-3-*p*-tolylpropan-1-one (Shobeiri *et al.*, 2011) has been investigated. Here, we report the synthesis and crystal structure of the title molecule, [4-Cl—C_6_H_4_]CH[NHC_6_H_4_-4-Br][CH_2_C(O)C_6_H_5_]. The asymmetric C atom has a slightly distorted tetrahedral configuration (Fig 1) with the bond angles in the range of 107.92 (16)° [N(1)—C(9)—C(8)] to 114.69 (16)° [N(1)—C(9)—C(10)]. In the crystal, pairs of intermolecular N—H···O(C) hydrogen bonds (Table 1) form centrosymmetric dimers as *R*_2_^2^(12) rings (for graph-set notation, see Bernstein *et al.*, 1995). A view of crystal packing is shown in Fig. 2.

### Experimental

To a magnetically stirred mixture of 3-(4-chlorophenyl)-1-phenylprop-2-en-1-one (0.24 g, 1.0 mmol) and Ag_3_PW_12_O_40_ (0.32 g, 0.10 mmol) as catalyst, in ethanol (5 ml), 4-bromoaniline (0.20 g, 1.2 mmol) was added at room temperature. The reaction completion was monitored by thin layer chromatography (TLC). The catalyst Ag_3_PW_12_O_40_ was collected by centrifugation. The reaction mixture was extracted with distilled water and ether (2×10 ml). The combined organic layer was evaporated to obtain crude product which was washed with hexane to give pure product. Single crystals of the product were obtained from a solution of CHCl_3_/CH_3_OH at room temperature.

### Refinement

H atoms of N—H was found in a difference Fourier map and refined isotropically with a distance restraint of N1—H = 0.81 (3) Å. The other H atoms were positioned geometrically and refined as riding atoms, with C—H = 0.93(aromatic CH), 0.97(CH2) and 0.98 (aliphatic CH) Å and with *U*_iso_(H) = 1.2 and 1.5 *U*_eq_(C).

### Crystal data

**Table:**

C_21_H_17_BrClNO	*F*(000) = 840
*M**_r_* = 414.72	*D*_x_ = 1.506 Mg m^−^^3^
Monoclinic, *P*2_1_/*n*	Mo *K*α radiation, λ = 0.71073 Å
Hall symbol: -P 2yn	Cell parameters from 9986 reflections
*a* = 10.6571 (4) Å	θ = 2.4–23.8°
*b* = 17.2432 (6) Å	µ = 2.40 mm^−^^1^
*c* = 10.8602 (4) Å	*T* = 296 K
β = 113.571 (2)°	Irregular, colorless
*V* = 1829.19 (12) Å^3^	0.35 × 0.31 × 0.11 mm
*Z* = 4	

### Data collection

**Table:**

Bruker APEXII CCD diffractometer	3983 independent reflections
Radiation source: fine-focus sealed tube	3274 reflections with *I* > 2σ(*I*)
graphite	*R*_int_ = 0.036
φ and ω scans	θ_max_ = 27.0°, θ_min_ = 2.3°
Absorption correction: multi-scan (*SADABS*; Sheldrick, 2004)	*h* = −13→13
*T*_min_ = 0.589, *T*_max_ = 0.746	*k* = −22→22
69312 measured reflections	*l* = −13→13

### Refinement

**Table:**

Refinement on *F*^2^	Primary atom site location: structure-invariant direct methods
Least-squares matrix: full	Secondary atom site location: difference Fourier map
*R*[*F*^2^ > 2σ(*F*^2^)] = 0.033	Hydrogen site location: inferred from neighbouring sites
*wR*(*F*^2^) = 0.084	H atoms treated by a mixture of independent and constrained refinement
*S* = 1.03	*w* = 1/[σ^2^(*F*_o_^2^) + (0.0314*P*)^2^ + 1.4336*P*] where *P* = (*F*_o_^2^ + 2*F*_c_^2^)/3
3983 reflections	(Δ/σ)_max_ = 0.001
230 parameters	Δρ_max_ = 0.71 e Å^−^^3^
0 restraints	Δρ_min_ = −0.86 e Å^−^^3^

### Special details

**Table:**

Geometry. All s.u.'s (except the s.u. in the dihedral angle between two l.s. planes) are estimated using the full covariance matrix. The cell s.u.'s are taken into account individually in the estimation of s.u.'s in distances, angles and torsion angles; correlations between s.u.'s in cell parameters are only used when they are defined by crystal symmetry. An approximate (isotropic) treatment of cell s.u.'s is used for estimating s.u.'s involving l.s. planes.
Refinement. Refinement of *F*^2^ against ALL reflections. The weighted *R*-factor *wR* and goodness of fit *S* are based on *F*^2^, conventional *R*-factors *R* are based on *F*, with *F* set to zero for negative *F*^2^. The threshold expression of *F*^2^ > 2σ(*F*^2^) is used only for calculating *R*-factors(gt) *etc*. and is not relevant to the choice of reflections for refinement. *R*-factors based on *F*^2^ are statistically about twice as large as those based on *F*, and *R*- factors based on ALL data will be even larger.

### Fractional atomic coordinates and isotropic or equivalent isotropic displacement parameters (Å^2^)

**Table:**

	*x*	*y*	*z*	*U*_iso_*/*U*_eq_	
Br	−0.04722 (3)	0.80334 (2)	−0.19048 (3)	0.06568 (12)	
Cl	−0.16806 (9)	0.56173 (4)	0.49694 (7)	0.0721 (2)	
O1	0.17572 (18)	1.03398 (10)	0.6206 (2)	0.0634 (5)	
N1	0.08682 (19)	0.90054 (10)	0.38597 (18)	0.0371 (4)	
C18	−0.0066 (2)	0.83266 (15)	−0.0093 (2)	0.0450 (5)	
C17	0.0762 (3)	0.78643 (15)	0.0933 (2)	0.0494 (6)	
H17	0.1118	0.7409	0.0743	0.059*	
C16	0.1072 (2)	0.80736 (13)	0.2259 (2)	0.0450 (5)	
H16	0.1635	0.7757	0.2953	0.054*	
C21	0.0549 (2)	0.87508 (12)	0.2558 (2)	0.0351 (4)	
C9	0.1518 (2)	0.85036 (11)	0.5014 (2)	0.0341 (4)	
H9	0.2417	0.8351	0.5049	0.041*	
C8	0.1731 (2)	0.89759 (12)	0.6286 (2)	0.0385 (5)	
H8A	0.0848	0.9064	0.6323	0.046*	
H8B	0.2281	0.8673	0.7069	0.046*	
C7	0.2419 (2)	0.97478 (12)	0.6350 (2)	0.0389 (5)	
C6	0.3887 (2)	0.97840 (12)	0.65830 (19)	0.0358 (4)	
C5	0.4741 (2)	0.91441 (13)	0.6986 (2)	0.0405 (5)	
H5	0.4400	0.8672	0.7132	0.049*	
C4	0.6101 (2)	0.92042 (15)	0.7171 (2)	0.0498 (6)	
H4	0.6673	0.8774	0.7450	0.060*	
C3	0.6605 (2)	0.98986 (17)	0.6944 (3)	0.0556 (6)	
H3	0.7514	0.9934	0.7053	0.067*	
C20	−0.0291 (2)	0.92111 (13)	0.1491 (2)	0.0437 (5)	
H20	−0.0654	0.9667	0.1670	0.052*	
C19	−0.0594 (2)	0.90066 (15)	0.0179 (2)	0.0486 (5)	
H19	−0.1149	0.9323	−0.0520	0.058*	
C10	0.0717 (2)	0.77732 (11)	0.5009 (2)	0.0336 (4)	
C11	0.1383 (2)	0.70874 (13)	0.5539 (2)	0.0455 (5)	
H11	0.2335	0.7072	0.5888	0.055*	
C12	0.0668 (3)	0.64243 (14)	0.5562 (3)	0.0538 (6)	
H12	0.1129	0.5967	0.5926	0.065*	
C13	−0.0743 (3)	0.64527 (13)	0.5035 (2)	0.0460 (5)	
C14	−0.1434 (2)	0.71292 (14)	0.4522 (2)	0.0460 (5)	
H14	−0.2385	0.7144	0.4188	0.055*	
C15	−0.0702 (2)	0.77871 (12)	0.4507 (2)	0.0409 (5)	
H15	−0.1166	0.8246	0.4156	0.049*	
C2	0.5775 (3)	1.05403 (16)	0.6559 (3)	0.0565 (6)	
H2	0.6125	1.1010	0.6417	0.068*	
C1	0.4420 (2)	1.04882 (13)	0.6382 (2)	0.0463 (5)	
H1	0.3862	1.0924	0.6129	0.056*	
H	0.031 (3)	0.9290 (15)	0.396 (3)	0.046 (7)*	

### Atomic displacement parameters (Å^2^)

**Table:**

	*U*^11^	*U*^22^	*U*^33^	*U*^12^	*U*^13^	*U*^23^
Br	0.04927 (16)	0.1060 (3)	0.03996 (14)	−0.00352 (14)	0.01597 (11)	−0.00835 (13)
Cl	0.0956 (5)	0.0517 (4)	0.0638 (4)	−0.0335 (4)	0.0266 (4)	0.0014 (3)
O1	0.0462 (10)	0.0427 (9)	0.0988 (15)	0.0070 (8)	0.0262 (10)	−0.0028 (9)
N1	0.0382 (9)	0.0331 (9)	0.0392 (9)	0.0026 (8)	0.0145 (8)	0.0021 (7)
C18	0.0366 (11)	0.0616 (14)	0.0372 (11)	−0.0081 (10)	0.0153 (9)	−0.0005 (10)
C17	0.0552 (14)	0.0516 (13)	0.0476 (13)	0.0056 (11)	0.0271 (11)	−0.0006 (11)
C16	0.0483 (12)	0.0469 (13)	0.0403 (11)	0.0105 (10)	0.0183 (10)	0.0080 (10)
C21	0.0319 (10)	0.0349 (10)	0.0376 (10)	−0.0048 (8)	0.0129 (8)	0.0024 (8)
C9	0.0296 (9)	0.0346 (10)	0.0368 (10)	0.0004 (8)	0.0117 (8)	0.0005 (8)
C8	0.0347 (10)	0.0433 (11)	0.0375 (11)	−0.0052 (9)	0.0144 (9)	−0.0027 (9)
C7	0.0371 (11)	0.0393 (11)	0.0370 (10)	−0.0010 (9)	0.0114 (9)	−0.0042 (9)
C6	0.0348 (10)	0.0375 (11)	0.0324 (10)	−0.0048 (8)	0.0106 (8)	−0.0037 (8)
C5	0.0361 (10)	0.0406 (11)	0.0395 (11)	−0.0032 (9)	0.0094 (9)	0.0001 (9)
C4	0.0366 (11)	0.0565 (14)	0.0495 (13)	0.0017 (10)	0.0100 (10)	−0.0039 (11)
C3	0.0380 (12)	0.0771 (18)	0.0495 (14)	−0.0132 (12)	0.0151 (10)	−0.0062 (13)
C20	0.0407 (11)	0.0390 (11)	0.0453 (12)	0.0026 (9)	0.0107 (10)	0.0040 (9)
C19	0.0398 (11)	0.0565 (14)	0.0406 (12)	0.0000 (10)	0.0068 (9)	0.0100 (10)
C10	0.0356 (10)	0.0316 (10)	0.0327 (9)	−0.0010 (8)	0.0127 (8)	−0.0009 (8)
C11	0.0405 (12)	0.0405 (12)	0.0484 (13)	0.0045 (9)	0.0104 (10)	0.0056 (10)
C12	0.0652 (16)	0.0361 (12)	0.0516 (14)	0.0019 (11)	0.0143 (12)	0.0081 (10)
C13	0.0624 (15)	0.0373 (12)	0.0386 (11)	−0.0157 (10)	0.0206 (11)	−0.0041 (9)
C14	0.0410 (12)	0.0478 (13)	0.0486 (13)	−0.0085 (10)	0.0173 (10)	−0.0052 (10)
C15	0.0363 (11)	0.0341 (10)	0.0493 (12)	0.0005 (8)	0.0141 (9)	0.0003 (9)
C2	0.0559 (15)	0.0589 (16)	0.0519 (14)	−0.0240 (13)	0.0186 (12)	0.0009 (12)
C1	0.0491 (13)	0.0408 (12)	0.0441 (12)	−0.0060 (10)	0.0134 (10)	0.0007 (10)

### Geometric parameters (Å, °)

**Table:**

Br—C18	1.906 (2)	C5—C4	1.385 (3)
Cl—C13	1.738 (2)	C5—H5	0.9300
O1—C7	1.215 (3)	C4—C3	1.374 (4)
N1—C21	1.386 (3)	C4—H4	0.9300
N1—C9	1.451 (3)	C3—C2	1.374 (4)
N1—H	0.81 (3)	C3—H3	0.9300
C18—C17	1.367 (3)	C20—C19	1.375 (3)
C18—C19	1.382 (4)	C20—H20	0.9300
C17—C16	1.391 (3)	C19—H19	0.9300
C17—H17	0.9300	C10—C11	1.381 (3)
C16—C21	1.387 (3)	C10—C15	1.388 (3)
C16—H16	0.9300	C11—C12	1.380 (3)
C21—C20	1.395 (3)	C11—H11	0.9300
C9—C10	1.520 (3)	C12—C13	1.379 (4)
C9—C8	1.540 (3)	C12—H12	0.9300
C9—H9	0.9800	C13—C14	1.373 (3)
C8—C7	1.507 (3)	C14—C15	1.381 (3)
C8—H8A	0.9700	C14—H14	0.9300
C8—H8B	0.9700	C15—H15	0.9300
C7—C6	1.483 (3)	C2—C1	1.381 (4)
C6—C5	1.385 (3)	C2—H2	0.9300
C6—C1	1.394 (3)	C1—H1	0.9300
			
C21—N1—C9	121.99 (17)	C3—C4—C5	120.0 (2)
C21—N1—H	115.5 (18)	C3—C4—H4	120.0
C9—N1—H	111.7 (18)	C5—C4—H4	120.0
C17—C18—C19	120.4 (2)	C2—C3—C4	120.4 (2)
C17—C18—Br	119.53 (19)	C2—C3—H3	119.8
C19—C18—Br	120.11 (17)	C4—C3—H3	119.8
C18—C17—C16	120.1 (2)	C19—C20—C21	121.5 (2)
C18—C17—H17	119.9	C19—C20—H20	119.3
C16—C17—H17	119.9	C21—C20—H20	119.3
C21—C16—C17	120.6 (2)	C20—C19—C18	119.5 (2)
C21—C16—H16	119.7	C20—C19—H19	120.3
C17—C16—H16	119.7	C18—C19—H19	120.3
N1—C21—C16	123.20 (19)	C11—C10—C15	118.5 (2)
N1—C21—C20	118.79 (19)	C11—C10—C9	120.90 (19)
C16—C21—C20	117.9 (2)	C15—C10—C9	120.61 (18)
N1—C9—C10	114.69 (16)	C12—C11—C10	121.4 (2)
N1—C9—C8	107.92 (16)	C12—C11—H11	119.3
C10—C9—C8	108.81 (16)	C10—C11—H11	119.3
N1—C9—H9	108.4	C13—C12—C11	118.8 (2)
C10—C9—H9	108.4	C13—C12—H12	120.6
C8—C9—H9	108.4	C11—C12—H12	120.6
C7—C8—C9	113.74 (17)	C14—C13—C12	121.2 (2)
C7—C8—H8A	108.8	C14—C13—Cl	118.75 (19)
C9—C8—H8A	108.8	C12—C13—Cl	120.06 (19)
C7—C8—H8B	108.8	C13—C14—C15	119.3 (2)
C9—C8—H8B	108.8	C13—C14—H14	120.4
H8A—C8—H8B	107.7	C15—C14—H14	120.4
O1—C7—C6	120.3 (2)	C14—C15—C10	120.9 (2)
O1—C7—C8	119.32 (19)	C14—C15—H15	119.6
C6—C7—C8	120.35 (18)	C10—C15—H15	119.6
C5—C6—C1	119.1 (2)	C3—C2—C1	120.0 (2)
C5—C6—C7	122.37 (19)	C3—C2—H2	120.0
C1—C6—C7	118.56 (19)	C1—C2—H2	120.0
C4—C5—C6	120.2 (2)	C2—C1—C6	120.2 (2)
C4—C5—H5	119.9	C2—C1—H1	119.9
C6—C5—H5	119.9	C6—C1—H1	119.9
			
C19—C18—C17—C16	0.4 (4)	C16—C21—C20—C19	−0.2 (3)
Br—C18—C17—C16	179.65 (18)	C21—C20—C19—C18	0.6 (3)
C18—C17—C16—C21	0.0 (4)	C17—C18—C19—C20	−0.7 (4)
C9—N1—C21—C16	−14.4 (3)	Br—C18—C19—C20	−179.93 (17)
C9—N1—C21—C20	168.47 (19)	N1—C9—C10—C11	145.8 (2)
C17—C16—C21—N1	−177.2 (2)	C8—C9—C10—C11	−93.3 (2)
C17—C16—C21—C20	−0.1 (3)	N1—C9—C10—C15	−36.2 (3)
C21—N1—C9—C10	−59.1 (2)	C8—C9—C10—C15	84.8 (2)
C21—N1—C9—C8	179.46 (17)	C15—C10—C11—C12	0.8 (3)
N1—C9—C8—C7	−50.4 (2)	C9—C10—C11—C12	178.8 (2)
C10—C9—C8—C7	−175.40 (17)	C10—C11—C12—C13	0.3 (4)
C9—C8—C7—O1	109.1 (2)	C11—C12—C13—C14	−1.5 (4)
C9—C8—C7—C6	−70.7 (2)	C11—C12—C13—Cl	176.77 (19)
O1—C7—C6—C5	168.4 (2)	C12—C13—C14—C15	1.5 (4)
C8—C7—C6—C5	−11.8 (3)	Cl—C13—C14—C15	−176.77 (18)
O1—C7—C6—C1	−11.9 (3)	C13—C14—C15—C10	−0.4 (3)
C8—C7—C6—C1	167.93 (19)	C11—C10—C15—C14	−0.7 (3)
C1—C6—C5—C4	−0.7 (3)	C9—C10—C15—C14	−178.8 (2)
C7—C6—C5—C4	179.0 (2)	C4—C3—C2—C1	−0.7 (4)
C6—C5—C4—C3	−0.6 (4)	C3—C2—C1—C6	−0.5 (4)
C5—C4—C3—C2	1.3 (4)	C5—C6—C1—C2	1.2 (3)
N1—C21—C20—C19	177.1 (2)	C7—C6—C1—C2	−178.5 (2)

### Hydrogen-bond geometry (Å, °)

**Table:**

*D*—H···*A*	*D*—H	H···*A*	*D*···*A*	*D*—H···*A*
N1—H···O1^i^	0.81 (3)	2.23 (3)	2.992 (3)	156 (2)

Symmetry codes: (i) −*x*, −*y*+2, −*z*+1.
